# Spectroscopic and Spectrometric Methods Used for the Screening of Certain Herbal Food Supplements Suspected of Adulteration

**DOI:** 10.15171/apb.2017.030

**Published:** 2017-06-30

**Authors:** Cristina Mateescu, Anca Mihaela Popescu, Gabriel Lucian Radu, Tatiana Onisei, Adina Elena Raducanu

**Affiliations:** ^1^National Office for Medicinal, Aromatic Plants and Bee Products - National Research and Development Institute for Food Bioresources – IBA Bucharest, 6 Dinu Vintila Str., 021102, Bucharest, Romania.; ^2^Faculty of Applied Chemistry and Materials Science - University Politehnica of Bucharest, 1-7 Polizu Str., 011061, Bucharest, Romania.

**Keywords:** Fourier transform infrared, GC-MS, Herbal food supplements, PDE-5 inhibitors, Raman spectroscopy

## Abstract

***Purpose:*** This study was carried out in order to find a reliable method for the fast detection of adulterated herbal food supplements with sexual enhancement claims. As some herbal products are advertised as "all natural", their "efficiency" is often increased by addition of active pharmaceutical ingredients such as PDE-5 inhibitors, which can be a real health threat for the consumer.

***Methodes:*** Adulterants, potentially present in 50 herbal food supplements with sexual improvement claims, were detected using 2 spectroscopic methods - Raman and Fourier Transform Infrared - known for reliability, reproductibility, and an easy sample preparation. GC-MS technique was used to confirm the potential adulterants spectra.

***Results:*** About 22% (11 out of 50 samples) of herbal food supplements with sexual enhancement claims analyzed by spectroscopic and spectrometric methods proved to be "enriched" with active pharmaceutical compounds such as: sildenafil and two of its analogues, tadalafil and phenolphthalein. The occurence of phenolphthalein could be the reason for the non-relevant results obtained by FTIR method in some samples. 91% of the adulterated herbal food supplements were originating from China.

***Conclusion:*** The results of this screening highlighted the necessity for an accurate analysis of all alleged herbal aphrodisiacs on the Romanian market. This is a first such a screening analysis carried out on herbal food supplements with sexual enhancement claims.

## Introduction

During the last period, the consumption of herbal food supplements meant to improve sexual performance has seen a considerable increase. Most consumers trust “100% Natural” advertised products considering them as safe and side effects free.^1^ In spite of consumers’belief, this category of herbal supplements could be "tainted" with legal drugs such as: sildenafil, tadalafil, vardenafil, as well as their analogues. Unfortunately, all these substances were not tested from pharmacological or pharmacokinetic point of view.^2,3^

Phosphodiesterase type 5 inhibitors (PDE-5 inhibitors), namely sildenafil, tadalafil, vardenafil are drugs commonly used to treat erectile dysfunction and can be consumed only on medical prescription.^4-6^ PDE-5 inhibitors are not recommended for patients on specific prescriptions as: organic nitrates (e.g. nitroglycerin, isosorbide dinitrate, isosorbide mononitrate, amyl nitrite, or nitrate used for the treatment of diabetes, hypertension, hyperlipidemia and ischemic heart disease), as they can cause serious and unpredictable blood pressure falls. These blood pressure falls are often accompanied by other specific symptoms among which: headache, flushing, dyspepsia, nasal congestion, dizziness, myalgia, back pain, and abnormal vision,are but a few to be mentioned.^7-9^

A number of efficient analytical methods such as: TLC, GC-MS, LC/MS/MS, LC-HR/MS, HPLC-DAD, HPLC-MS, NMR have been developed in order to detect the PDE-5 inhibitors adulteration of food supplements.^1,10-15^ Attia et al., have determined the vardenafil hydrocholorides as pure active ingredients in drugs using the TAI (thermal analysis investigation).^16^

However, all these methods require a laborius sample preparation and analysis while being very expensive from financial point of view. Therefore, for a quality screening of adulterated food supplements, a new approach was required: a non-destructive procedure, less laborious in respect of sample preparation and analysis, a faster and more efficient method involving minimal costs. Among such methods, Raman and Infrared spectroscopy proved to be the most efficient. According to specialized literature, these methods have been applied for various purposes: Kim et al. performed quality control of active pharmaceutical ingredients in drugs (capsules) using Raman spectroscopy;Olds used Spatially Offset Raman Spectroscopy (SORS) to identify concealed substances from a multi-layered postal package. Other identifications were performed for capsules with antibiotics in plastic blister packs and drug dissolved in clear solvents bottled in opaque plastic vials. De Veij et al., detected counterfeit Viagra tablets using Raman while Trefi et al., using Raman spectroscopy and NMR (Nuclear Magnetic Resonance) identified counterfeit Cialis tablets illegally sold on the internet.^17-20^ Fourier Transform Infrared Spectroscopy with Attenuated Total Reflectance (ATR-FTIR) and IR Spectroscopy were used to detect counterfeit drugs like Viagra and Cialis type by Ortiz et al., and Custers et al.^21,22^ Using the same techniques, Champagne et al., detected sildenafil and tadalafil in raw materials used as ingredients in food supplements.^23^ Chuang et al., and Yang et al., used near-infrared spectroscopy (NIR) to analyze bioactive compounds in herbs and herbal medicines, respectively.^24,25^

As consumers show an increasing demand for natural products (supplements), quality control, adequate risk assessment and clear regulation for botanicals and botanical preparations are highly required. While adulteration could be “economically motivated”, occurrence of such pharmacological active compounds in herbal supplements may become a serious health threat for consumers.

In this study, a qualitative screening of 50 herbal food supplements samples collected from the local market, was carried out. Raman spectroscopy and Fourier Transform Infrared Spectroscopy were used as rapid screening methods. These analytical methods are complementary and cheaper and can be used to identify several chemical functional groups. They are less time consuming in both sample preparation and analysis. As soon as the adulteration was detected, the GC-MS technique was applied to confirme the adulterants spectra in the respective samples.

## Materials and Methods

### Chemicals

Reference standards of sildenafil (with purity of 98,8%) and tadalafil (with purity of 99,9%) were purchased from European Directorate for the Quality of Medicines & HealthCare, European Pharmacopoeia (Strasbourg, France). Acetone and methanol used as solvents were provided by Merck, Germany.

### Commercial formulations of dietary supplements

A total number of 50 herbal food supplements promoted to improve sexual performance were analyzed. The samples were encoded from Hfsd1 up to Hfsd50. A number of 45 products were provided by the National Office for Medicinal, Aromatic Plants and Bee Products, 4 products were purchased on line while one product was bought from a local drugstore. The analyzed herbal supplements originated from different countries and were marketed as: capsules, tablets, liquids in vials, sachets, powders.

### Raman measurements

Raman spectra were recorded by a NXR FT-Raman Module with InGaAs (Inidium-Gallium Arsenide) detector and CaF_2_ beam splitter. The power of laser beam at the surface of the sample was about 0.3 mW. Each spectrum consisted of 64 co-added scans at a spectral resolution of 4 cm^-1^ in the field of 3701-100 cm^-1^. Omnic software version 8 (Nicolet Instrument Co. Madison, USA) was used to determine the spectra.

### ATR-FTIR measurements

A Nicolet 6700 FT-IR Spectrometer (Nicolet Instrument Co., Madison, USA) with DTGS (Deuterated Triglycine Sulphate) detector was used to record the absorbtion spectra.

A single bounce ZnSe – diamond crystal was used in the attenuated total reflectance (ATR) sampling system. A small amount of each homogenized sample was directly applied and pressed on the diamond crystal. The same pressure value was applied to all samples. Each spectrum was measured at a spectral resolution of 4 cm^-1^ and consisted of 64 co-added scans. Recordings were performed in the range of 3701-100 cm^-1^. The crystal was cleaned in acetone and dried in open air, at room temperature following each measurement. To avoid possible contamination of the crystal, the background spectrum using identical instrumental conditions was measured after each acetone cleaning.

### GC-MS analysis

For analytical screening and confirmation of the adulterated herbal food supplements, GC-MS was applied. The GC-MS system consisted of an Agilent HP6890N Gas Chromatograph and a HP5973N Mass Spectrometer. 1 µL volume was injected using splitless mode on the 250^o^ C injector port with helium flow at 1.0 mL/min. A TR-5MS (30 m x 0.25 mm i.d. 0.25 µm film thickness) capillary column (5% phenyl polysilphenylene-siloxane) was used. The interface temperature was set at 290^o^ C. The oven ramping temperature was programmed at 135-200^o^ C (1 min hold) at a rate of 13^o^C/min, and 200-315^o^C at a rate of 6^o^ C/min, at 315^o^ C holds for 20 min. The screening was performed on selected ion monitoring mode at *m/z* 99, 404 for sildenafil and *m/z* 389, 262 for tadalafil, respectively. Adulterant identification was done on full scan mode (50-500 a.m.u), Man et al., adapted method.^26^

### Sample preparation

The sample required minimal preparation. Each solid sample consisted of: the content of one capsule, a tablet (that was crushed) or the contents of a sachet. The powder obtained from each sample was homogenized using a mortar and pestle. For Raman analysis the obtained homogenized powder was inserted into a 6 mm diameter vial, that was further inserted in the equipment.

Liquid samples did not require previously preparation.

### GC-MS analysis

#### Stock solutions of sildenafil and tadalafil

For stock solutions preparation, 1 mg of high purity reference standards (sildenafil and tadalafil, respectively) was each dissolved in 1 ml absolute methanol. To get the reference ion chromatogram for each adulterant, a 1:10 dilution was performed.

#### Samples

Each 100 mg of homogene fine powder/sample (from a sachet, by emptying a capsule or crushing a tablet) was disolved in 1 ml of absolute methanol. For the liquid products, 2 ml/sample were taken and dilluted in 1 ml absolute methanol. Samples were thoroughly vortexed, followed by 15 minutes of sonication and 5 minutes centrifugation at 4000 rpm. The supernatant was collected and filtered by 0,2 µm membrane filters for GC-MS analysis.

## Results and Discussion

### Raman spectroscopy

The high purity reference standards of sildenafil and tadalafil were analyzed.

The specific bands corresponding to the characteristic functional groups of sildenafil were identified at 1698 cm^-1^ (band that can be attributed to stretching vibrations of the group C=O) as well as at the doublet 1580/1563 cm^-1^ (which is specific to C=C bond). For bonds containing nitrogen, Raman bands were present at 1529 cm^-1^, due to υ(N-C=N) and at 1238 cm^-1^, respectively, due to υ(C=N). Raman bands registered at 1170 cm^-1^ and 648 cm^-1^ are attributed to the symmetrical group (SO2), as well as to the stretching vibrations υ(C-S), respectively.^19^

Specific responses for tadalafil occurred in the 3100-3000 cm^-1^ range and 1700-1500 cm^-1^ range, respectively. The characteristic spectral bands are consistent with the literature data.^20^ These bands correspond to the vibrations of unsaturated or aromatic C-H bond and to the vibrations of unsaturated C=C bond, respectively.

The Raman spectra for all the 50 samples of dietary supplements that were registered according to the same procedure used for the reference standards.

As seen in Table 1 (A), four herbal supplements adulterated with sidenafil were identified. Almost all characteristic bands of high purity reference standard were present in the spectra of Hfsd50 sample (1170 cm^-1^ band was missing) and Hfs48 sample (1238 cm^-1^ was missing), while two or three bands were absent in the spectra of Hfsd4 and Hfsd49 samples.

As shown in Table 1 (B), other seven samples of herbal food supplements adulterated with tadalafil were also detected. Hfsd30 sample showed all the characteristic spectral bands of tadalafil, while in the spectra of Hfsd12 and Hfsd29 samples, one specific band was missing. In the spectra of Hfsd27 and Hfsd32 samples two and three bands, respectively, were missing. It has also been noticed that the spectra of the examined samples were of poor quality.

**Table 1:** Characteristic Raman bands of sildenafil and tadalafil detected in the analyzed samples

**(A) Pure Sildenafil - reference standard/** **Wave number (cm**^-1^)	**Sample**						
	**Hfsd4**	**Hfsd48**	**Hfsd49**	**Hfsd50**	**-**	**-**	**-**
1698 1580 1563 1529 1238 1170 648	1689 1583 1555 - - 1175 632	1682 1595 - 1524 1237 1164 652	- 1579 1566 1539 - - 648	1701 1580 1563 1529 1239 - 647			
**(B) Pure Tadalafil – reference standard/** **Wave number (cm**^-1^)	**Sample**						
	**Hfsd12**	**Hfsd27**	**Hfsd28**	**Hfsd29**	**Hfsd30**	**Hfsd31**	**Hfsd32**
3066 3002 1672 1596 1567	3056 - 1685 1592 1544	3046 - 1697 - 1567 1528 1514	3055 - - 1591 -	3098 3028 - 1597 1566	3095 3074 3057 1643 1611 1580 1564	- - - 1594 1571 1520	3066 - - 1591 - 1525

In the Hfsd28 and Hfsd31 samples spectra only a part of the characteristic bands of tadalafil were detected. As the Raman spectra of the Hfsd28 and Hfsd31 samples were not enough relevant a clear conclusion on their tadalafil adulteration could not be drawn.

Raman spectroscopy used to screen the 50 herbal food supplements with sexual enhancement claims, detected 9 adulterated samples among which 4 products with sildenafil and 5 products with tadalafil. As already mentioned, two samples suspected to be adulterated with tadalafil showed no relevant spectra, missing three specific bands of the reference standard.

### ATR-FTIR spectroscopy

The FTIR spectra for the 50 samples of herbal food supplements were also analysed against the sildenafil and tadalafil reference standards.

Absorption peaks characteristic for PDE-5 inhibitors were registerd in the 1800-525 cm^-1^ range. According to Champagne et al., this spectral range includes the 1720-1150 cm^-1^domain, important for the detection of PDE-5 inhibitors analogues and homologues, respectively.^23^

**Figure 1:**
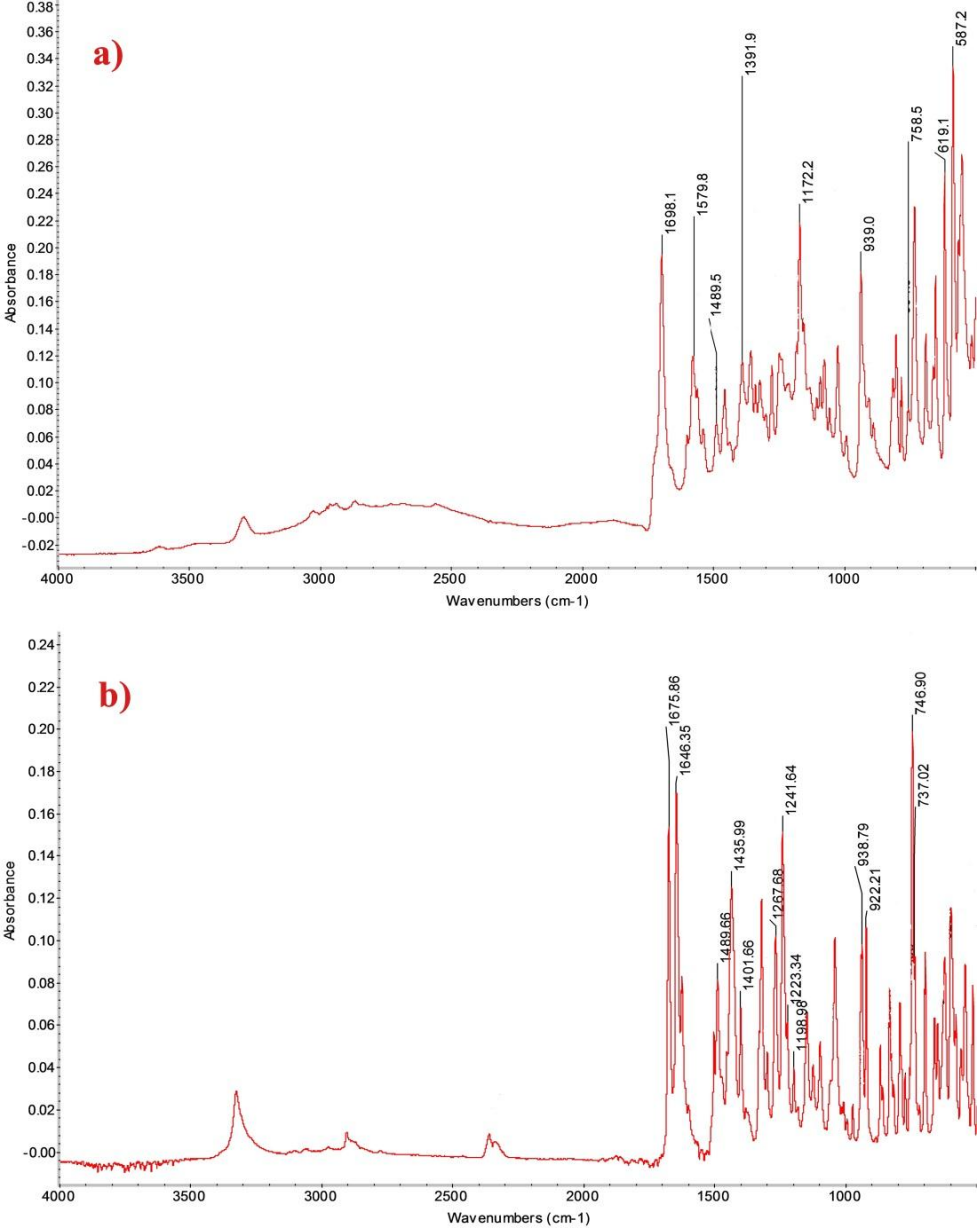
FTIR spectra for high purity reference substances: a) sildenafil and b) tadalafil

The sildenafil spectrum (Figure 1a) showed significant absorption peaks at: 1698 cm^-1^ (characteristic to carbonyl groups (C=O)); 1579 cm^-1^ (specific to N-H bonds, occurring in the range of 1650-1500 cm^-1^); 1489 cm^-1^ (resulted from C=C bonds in the benzene ring). C-N bonds from the functional group O=C-N is absorbed at 1400 cm^-1^ (but in this experiment the absorbtion value was 1391 cm^-1^). Anzanello et al. reported that C-H aromatic out-of-plane deformation occurred at 939 cm^-1^, which resulted in addition of new peaks at 1172, 758, 619, 587 cm^-1^.^27^

The specific tadalafil absorption peaks of FTIR spectrum (Figure 1b) were registered at 1675 cm^-1^ (characteristic of amides C=O), 1646 cm^-1^ (C=C aromatic). The band of 1435 cm^-1^ belongs to the stretching vibration C-N, and the band 746 cm^-1^ is representative for benzene.^27^

Comparing the spectrum of sildenafil reference standard with all spectra of the 50 analyzed samples of herbal food supplements, the occurence of sildenafil adulteration of four samples (Hfsd4, Hfsd48, Hfsd49, Hfsd50) was noted (Table 2 (A)).

All characteristic absorption peaks for sildenafil were identified in the spectrum of Hfsd50 sample. The Hfsd48 sample spectrum did not show, the characteristic absorption peaks for: 1579 cm^-1^, 1172 cm^-1^, and 758 cm^-1^. In the spectrum of Hfsd49 sample the four bands: 1579 cm^-1^, 1489 cm^-1^, 1391 cm^-1^ and 758 cm^-1^, respectively, were absent. Hfsd4 sample showed a very poor spectrum (6 bands were missing: 1698 cm^-1^, 1391 cm^-1^, 1172 cm^-1^, 758 cm^-1^, 619 cm^-1^ and 587 cm^-1^, respectively). This last result is not relevant if the adulteration with sildenalfil is to be considered.

Out of the total analyzed samples, seven herbal food supplements (Hfsd12, Hfsd27, Hfsd28, Hfsd29, Hfsd30, Hfsd31, Hfsd32) were identified as adulterated with tadalafil (Table 2 (B)). All spectra of the adulterated samples showed the same absorption peaks as the tadalafil reference spectrum. Thus, FTIR analysis confirmed that Hfsd28 sample was adulterated with tadalafil while application of Raman spectroscopy to the mentioned sample could not detect this adulteration.

The screening of 50 herbal food supplements with sexual enhancement claims performed by ATR-FTIR spectroscopic method had as result the detection of a number of 10 adulterated samples with sildenafil (3 products) and tadalafil (7 products).

**Table 2:** Characteristic FTIR bands of sildenafil and tadalafil detected in the analyzed samples

**(A) Pure Sildenafil - reference standard/ Wave numbers (cm** ^ 1 ^ )	**Sample**						
	**Hfsd4**	**Hfsd48**	**Hfsd49**	**Hfsd50**	**-**	**-**	**-**
1698 1579 1489 1391 1172 939 758 619 587	- 1580 1463 - - 934 - - -	1687 - 1488 1397 – 932 – 617 583	1698 - - - 1172 939 - 619 588	1700 1581 1490 1392 1171 939 758 619 588			
**(B) Pure Tadalafil – reference standard/ Wave numbers (cm** ^1^)	**Sample**						
	**Hfsd12**	**Hfsd27**	**Hfsd28**	**Hfsd29**	**Hfsd30**	**Hfsd31**	**Hfsd32**
1675 1646 1435 746	1674 1645 1433 746	1675 1646 1436 745	1675 1646 1436 745	1675 1646 1435 747	1675 1646 1436 746	1675 1645 1435 746	1675 1648 1435 748

### GC-MS

Due to its capacity to separate, quantify and identify unknown organic compounds, GC-MS was used as a sensitive method to confirm the results obtained by the two spectroscopic techniques (Raman and FTIR). The advantage of this method is related to the sample preparation (no derivatization or hydrolysis procedure is needed) as well as to the detection time (much shorter) of adulterant substances.

A good chromatographic separation of sildenafil and tadalafil reference standards was obtained at a retention time of 38.66 minutes, 36.63 minutes, respectively. Identification of the two PDE-5 inhibitors (namely sildenafil and tadalafil) by GC-MS was facilitated by the presence of their molecular ion *m/z* 474 (for sildenafil) and *m/z* 389 (for tadalafil).

Out of the total number of analyzed samples, 11 herbal food supplements proved to be adulterated with sildenafil or tadalafil (Table 3). The adulterated products (91% of the analyzed samples) were of Chinese origin.

**Figure 2:**
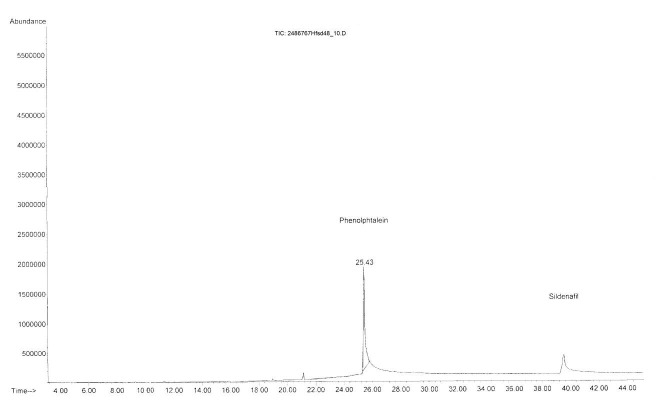
Total ion chromatogram of Hfsd48 sample adulterated with sildenafil and phenolphthalein

Surprisingly, phenolphthalein was detected in Hfsd48 sample (Figure 2), in which sildenafil was also detected. It has to be noticed that phenolphthalein is a banned substance both in Europe and in USA since 1997 because of its potential carcinogenic effects. We suppose that phenolphthalein was introduced in the herbal food supplement with the intention to conceal the presence of sildenafil and thus to prevent the detection of the adulterant (PDE-5 inhibitor) by spectroscopic analysis.

The occurence of phenolphthalein could be the reason for the non-relevant results obtained by FTIR method in Hfsd48 sample.

**Figure 3:**
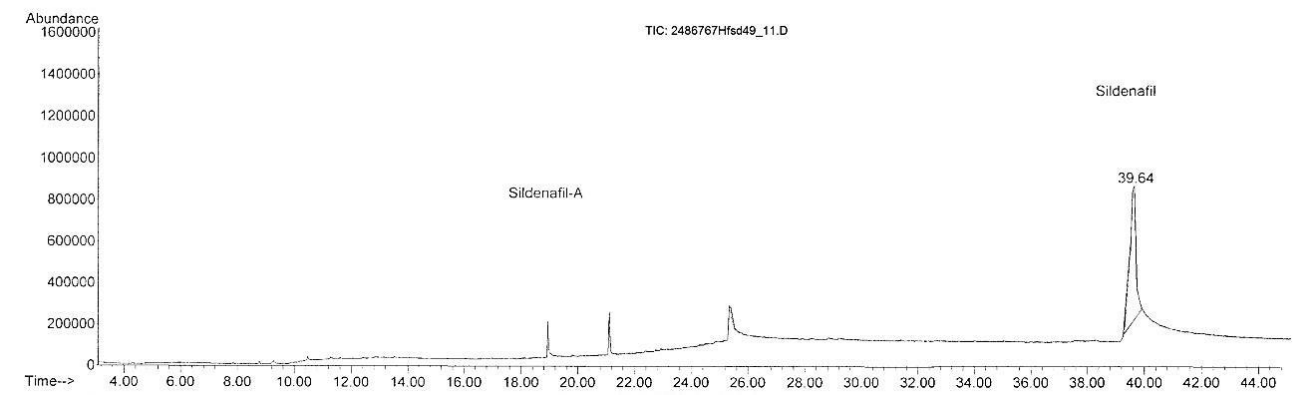
Total ion chromatogram of Hfsd49 sample adulterated with sildenafil and two similar compounds

GC-MS technique proved to be more sensitive than spectroscopic methods Raman and ATR-FTIR: in Hfsd4, Hfsd49 and Hfsd50 samples, sildenafil was identified togeher with other two unknown compounds with similar fragmentation pattern (probably analogues of sildenafil) as it could be seen in Figure 3.

We suppose that sildenafil analogues (detected in Hfsd4, Hfsd49 and Hfsd50 samples) as well as phenolphthalein (detected in Hfsd48 sample) identified by GC-MS could be responsible for the low quality spectra recorded by ATR-FTIR method used to screen the herbal food supplements.

**Figure 4:**
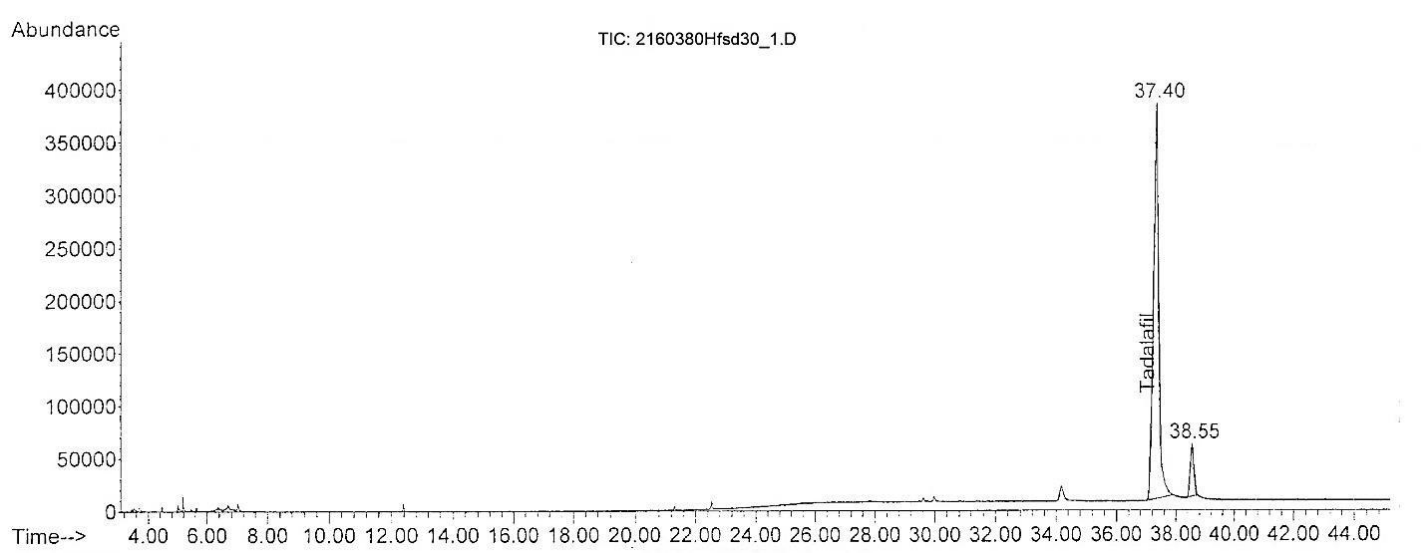
Total ion chromatogram of Hfsd30 sample adulterated with tadalafil

Using GC-MS analysis, the adulterant tadalafil was detected in seven herbal food supplements (Hfsd12, Hfsd27, Hfsd28, Hfsd29, Hfsd30, Hfsd31, and Hfsd32 samples). The chromatograms of all samples showed a common significant peak for tadalafil at the retention time at 37.4 min (see Figure 4).

**Table 3:** Characterization of analyzed herbal food supplements

**Sample**	**Product description**	**Country of origin**	**Identified adulterants**			**Analytical methods used**		
			**Sildenafil**	**Tadalafil**	**Others**	**Raman**	**FT-IR**	**GC-MS**
Hfsd 4	flask with 60 capsules	China	+	-	Two analogues	+	Not relevant spectrum	+
Hfsd 12	flask with 15 capsules	China	-	+	-	+	+	+
Hfsd 27	Box of 1 blister with 4 capsules	China	-	+	-	+	+	+
Hfsd 28	Box of 1 blister with 4 capsules	China	-	+	-	Not relevant spectrum	+	+
Hfsd 29	Box of 1 blister with 4 capsules	China	-	+	-	+	+	+
Hfsd 30	Box of 1 blister with 4 capsules	China	-	+	-	+	+	+
Hfsd 31	Box of 1 blister with 4 capsules	China	-	+	-	Not relevant spectrum	+	+
Hfsd 32	Box of 1 blister with 4 capsules	China	-	+	-	+	+	+
Hfsd 48	Box of 1 blister with 2 capsules	USA	+	-	Phenolphthalein	+	+	+
Hfsd 49	Foil with 2 capsules	China	+	-	Two analogues	+	+	+
Hfsd 50	Box of 1 blister with 4 tablets	China	+	-	Two analogues	+	+	+

The GC-MS method used to confirm the results of the spectroscopic methods applied for same samples showed that tadalafil could be detected more specific as compared to Raman technique (Hfsd28 and Hfsd31 samples were relevant examples).

About 22% of herbal food supplements with sexual enhancement claims analyzed by spectroscopic and spectrometric methods proved to be "enriched" with active pharmaceutical compounds such as: sildenafil and two of its analogues, tadalafil and phenolphthalein.

All these adulterants were detected in similar alleged herbal aphrodisiacs by different researchers (see literature data).^13-15^

## Conclusion

The screening performed on a total number of 50 herbal food supplements promoted as natural aphrodisiacs emphasised that 11 samples (22% of the analyzed products) were adulterated with pharmaceuticals compounds (PDE-5 inhibitors and their analogues) or chemical substances (phenolphthalein).

The adulterated food supplements are a real health threat for the consumers, due to the misleading labelling (“100% natural products”) and undeclared pharmaceutical substances hidden in their composition. The consumption of these adulterated herbal food supplements can seriously harm health as PDE-5 inhibitors and their analogues the most frequent adulterants in sexual enhancement products) could interact with nitrates based drugs and result in side effects or adverse reactions. As adulteration could affect not only the safety of end products but also the raw materials used by manufacturers, the fast screening spectroscopic methods could have more applications in the market control and surveillance of natural products.

In this study, the detection of adulterants using spectroscopic methods (Raman and Fourier transform infrared) was confirmed by gas chromatography coupled with mass spectrometry (GC-MS).

The minimal sample preparation required by spectroscopic methods as well as the very short analysis time (about 5 minutes/sample) and minimal costs (no special reagents are needed) are the main advanteges when a huge number of samples have to be screened. Thus, spectroscopic methods proved to be a very useful tool for the control and surveillance of the herbal food supplements market.

The screening of the products suspected to be adulterated could be rapidly performed. As soon as non-compliant products are identified, the adulterants can be detected by GC-MS techniques, which proved to be a very sensitive key method able to confirm the spectroscopic results.

The preliminary results obtained by the screening of 50 herbal food supplements with sexual enhancement claims, highlighted the necessity for an accurate analysis of all alleged herbal aphrodisiacs commercialized on the Romanian market, due to their potential risk profile.

## Acknowledgments

The work has been funded by the Sectoral Operational Programme Human Resources Development 2007-2013 of the Romanian Ministry of Labour, Family and Social Protection through the Financial Agreement POSDRU/107/1.5/S/76903.

## Ethical Issues

Not applicable.

## Conflict of Interest

The authors declare no conflict of interests.
